# Janus Charged Droplet Manipulation Mediated by Invisible Charge Walls

**DOI:** 10.1002/advs.202204382

**Published:** 2022-10-06

**Authors:** Qiangqiang Sun, Xuanming Hu, Boran Xu, Shiji Lin, Xu Deng, Shaobing Zhou

**Affiliations:** ^1^ Key Laboratory of Advanced Technologies of Materials Ministry of Education School of Materials Science and Engineering Southwest Jiaotong University Chengdu 610031 China; ^2^ School of Physics University of Electronic Science and Technology of China Chengdu 610054 China; ^3^ Institute of Fundamental and Frontier Sciences University of Electronic Science and Technology of China Chengdu 610054 China

**Keywords:** droplet manipulation, electrostatic induction, superhydrophobic, surface charge

## Abstract

The ability to control the mobility and function of droplets is fundamental to developing open surface microfluidics. Despite notable progress in the manipulation of droplets, the existing strategies are still limited in functionalizing droplets. Herein, the coupling of droplet motion and functionalization elicited by an invisible charge wall is reported. The charged superamphiphobic surface is overlapped with a conductor to induce free charge, creating the invisible charge wall at the overlapping boundary. The charge wall can trap droplets and polarize them into Janus charged state. It is found that the trapping degree and the charge distribution in the Janus charged droplet depend on the original surface charge on the superamphiphobic surface. The invisible charge wall can also be established at diverse boundary curvatures, allowing to design pathways for droplet manipulations. Furthermore, the enrichment of protein and nanomaterial in the manipulated Janus charged droplet is demonstrated. The strategy provides a potential microfluidic platform with orthogonal functionalities.

## Graphical abstract

Controlling the mobility and function of droplets is fundamental to developing open surface microfluidics. Invisible charge walls are designed for droplet manipulation and for producing Janus charged droplets. Charging the droplet with Janus property enables to enrich and separate the composite in droplets. This invisible charge wall provides a versatile microfluidic platform with orthogonal functionalities.

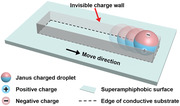

## 1 Introduction

Droplet manipulation is crucial for many applications such as chemical reactions,^[^ ^1^ ^]^ bio‐analysis,^[^ ^2^, ^3^ ^]^ energy harvesting, and drug delivery.^[^ ^4^, ^5^, ^6^, ^7^ ^]^ The control of droplet motion is extensively investigated by various strategies, including body force,^[^ ^8^ ^]^ electricity,^[^ ^9^, ^10^, ^11^, ^12^ ^]^ mechanics,^[^ ^13^, ^14^, ^15^ ^]^ magnetism,^[^ ^6^, ^16^, ^17^, ^18^ ^]^ light,^[^ ^19^, ^20^, ^21^, ^22^ ^]^ wettability gradient,^[^ ^23^, ^24^, ^25^ ^]^ and electrostatic charge.^[^ ^26^, ^27^, ^28^ ^]^ Typically, the manipulated droplet acts as a carrier or container for matter and energy. The property of droplet itself has a remarkable influence on chemical, biological, and engineering applications, considering the transfer of matter and energy.^[^ ^29^, ^30^, ^31^, ^32^, ^33^, ^34^ ^]^ However, current approaches to manipulating droplets mainly focus on the droplet dynamics, while ignoring the regulation of chemical and physical properties in droplets. This kind of regulation refers to controlling the nature of the droplet itself such as polarity and charge, rather than introducing other components like magnetic particles into droplets. The property control of droplets can help to realize their functions, avoiding external interference at the same time. Therefore, coupling the mobility and functionalization of droplets is critical for developing the next generation of open surface microfluidic platforms, but has received rare attention.

Here, we develop an invisible charge wall enabling transport and functionalization of droplets on superamphiphobic surface. Superamphiphobic surface is a kind of superwetting surface that is simultaneously superhydrophobic and superoleophobic.^[^ ^35^ ^]^ Specially, we fabricate a small conductor film underneath the superamphiphobic surface, on which surface energy and topography would not be changed. One point of the superamphiphobic surface in the overlapping area is charged by modulating water droplet dynamics. The generated surface charge is stationary and induces free charge within the conductive substrate to establish an invisible charge wall at the overlapping boundary. The invisible charge wall can obstruct the droplet to roll across and polarize the droplet to become Janus charged. By designing the shape of the overlapping boundary, the Janus charged droplet can be transported along with the invisible charge wall. The two sides of Janus charged droplet carry opposite charges, generating dipole inside the droplet. This endogenous charge makes Janus charged droplet great potential value in many fields such as asymmetric synthesis, components separation, self‐assembly and biosensing, which cannot be realized by single‐attribute properties.

## 2 Results and Discussion

### 2.1 Design of Invisible Charge Walls

**Figure 1:**
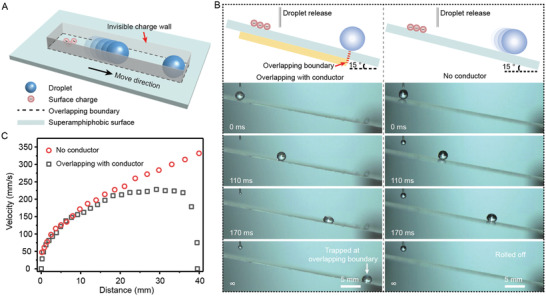
Droplet trapping mediated by an invisible charge wall. A) A sketch of the concept of invisible charge wall. The conductive film is pasted below a charged superamphiphobic surface. The overlapping boundary acts as an invisible charge wall to stop the motion of rolling droplet. B) The schematic and sequence of different motion states of the droplet on charged superamphiphobic surface in (left) and out (right) of contact with a conductor. The superamphiphobic surface is fabricated on 170 µm‐thick glass slide. C) The variation of droplet velocity as a function of advancing distance.

**Figure** **1**A shows the sketch of an invisible charge wall to stop motion of droplet on a superamphiphobic surface, which is prepared by candle soot template.^[^ ^35^ ^]^ The invisible charge wall is fabricated at the overlapping boundary of charged superamphiphobic surface and conductive film. The superamphiphobic surface is preferentially charged by water impact at a random position in the overlapping area. The amount of surface charge (*Q*) can be determined by Weber number (*We*), *Q*∝*We* ^0.74^.^[^ ^26^ ^]^ Here, *We* is a dimensionless number which is the ratio of the kinetic energy to the surface energy. We observed different behaviors of free droplets on the charged superamphiphobic surface with same wettability and inclined angle (Figure 1B and Movie S1, Supporting Information). The invisible charge wall can be effectively established when the superamphiphobic surface is prepared by materials with low relative permittivity. The superamphiphobic surface used in this research is fabricated with a dielectric substrate of 170 µm‐thick glass. A droplet rolls down the surface not in contact with conductor, while another droplet is trapped on the surface pasted with a conductive film. We plot the velocity of both droplets and clearly show the changing process of rolling and trapping (Figure 1C and Figure S1, Supporting Information). For the roll‐off droplet, we assume its velocity is determined by the gravitational driving force and inclined angle *α*, regardless of the frictional (viscous) dissipation. For the trapped droplet, it feels the presence of resistance force. We infer that the generated surface charge on superamphiphobic surface induces negative charges at the boundary of conductor to stop the motion of droplet. The charges accumulated at the conductor boundary confine the droplet motion in the overlapping area, acting as an invisible wall for the rolling droplet.

### 2.2 Charging Droplets with Janus Property

**Figure 2:**
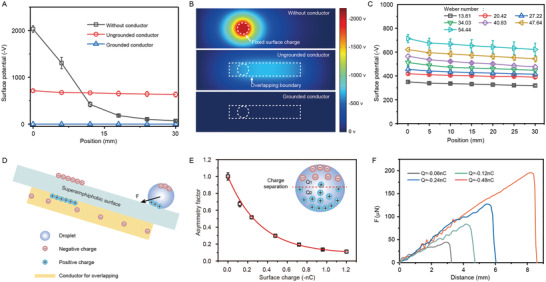
The mechanism of trapping and generating Janus charged droplet mediated by the invisible charge wall. A) The distribution of surface potential on charged superamphiphobic surface contacting without conductor, with conductor, and with grounded conductor, as a function of position. B) The simulation of the surface potential on the charged superamphiphobic surface corresponding to (A). C) Surface potential on the charged superamphiphobic surface contacting with ungrounded conductor under different Weber number of water drop. D) A schematic showing the distribution of charges in the overlapping conductor and in the droplet. E) The asymmetry of Janus charged droplet as a function of surface charge on the superamphiphobic surface. The top (*Q*_1_) and bottom (*Q*_2_) of droplet are divided with equal volume for charge measurements. The ratio of *Q*_1_ over *Q*_2_ is the asymmetry factor. Inset: Schematic diagram of Janus charged droplet. F) The trapping force under different surface charge, measured by a homemade set‐up (Note S3, Supporting Information).

To investigate how the conductor influences the droplet dynamics, we measured the surface potential in the overlapping area in one‐dimensional direction (**Figure** **2**A). We find that the conductor significantly changed the distribution of surface potential, resulting from the rearrangement of charge in conductor. The effect of conductor on the distribution of surface potential is also proved by the simulation work (Figure 2B, Note S1, Supporting Information). This result suggests that the original negative surface charge (*Q*) on superamphiphobic surface generated by water impact is stationary, which induces opposite positive charge in conductor. Since the conductor is neutral as a whole, the equivalent movable negative charges (*Q*_m_ *=* *Q*) in conductor are rearranged to reach electrostatic balance. The movable free charge can also be discharged if the conductor is grounded, resulting to complete screening of surface charge. We consider the amount of movable free charge on conductor is equivalent to the original surface charge based on the principle of electrostatic induction.^[^ ^36^ ^]^ Thus, the movable free charge is proportional to

(1) $$Q_{m}\propto We^{0.74}$$

This relationship is confirmed by the surface potential measurement varying the Weber number of impacting water (Figure 2C).

The free charge is the basis of forming charge wall to restrict mobility of droplets at the overlapping boundary. Its distribution depends on the boundary curvature of conductor in an isolated system.^[^ ^37^, ^38^ ^]^ Here, we consider the pasted conductor as two‐dimensional circle, owing to the thin thickness. The charge density (*σ*) at a point on conductor is expressed as $\sigma =\frac{Q_{m}}{\pi r^{2}}$, where *r* is the radius of the circular conductor. The mean curvature (*κ*) of the conductor boundary is written as $\kappa =\frac{1}{r}$. Thus, the charge density at the overlapping boundary is positively correlated to the boundary curvature and original surface charge: *σ* ≈ *κ* ^2^ *Q*. The accumulated charges at the boundary becomes the invisible charge wall. Unsimilar to conventional defect of chemical or topographic patterns, the invisible charge wall can polarize the droplet, generating a Janus charged droplet which owns asymmetrical charge distribution. Figure 2D shows the formation of invisible charge wall and Janus charged droplet. Charging droplet and Janus property are both of intrinsic interest and of practical value as a chemical reactor for synthesis and catalysis.^[^ ^28^, ^39^, ^40^, ^41^, ^42^ ^]^ The development of Janus charged droplet provides great potential in chemistry, and bioassays. We characterized the Janus charged droplet and found that the asymmetry of Janus charged droplet depends on the original surface charge generated on superamphiphobic surface (Figure 2E and Figure S2, Supporting Information). The adjustability enables us to tune the asymmetry of Janus charged droplets based on the requirements of applications.

We continue to discuss the electric force exerted by the invisible charge wall on the trapped droplet. Assuming that the charge of droplet before releasing is neutral, which will not change the free charge distribution when contacting on the superamphiphobic surface. The charge wall produces an inhomogeneous electric field and polarizes the droplet, leading to a net electric force. Based on the electrical analysis, the electric force associated with the Janus charged droplet can be expressed as

(2) $$F∼\left(\varepsilon_{r}-1\right)\varepsilon_{0}\kappa^{4}Q^{2}$$

where *ε*_r_ is the relative dielectric constant of the droplet, *ε*_0_ is the dielectric constant of empty space (Note S2, Supporting Information).^[^ ^26^, ^43^ ^]^ We also directly measured the lateral resistance force by a homemade set‐up (Note S3, Supporting Information). The result shows that the trapping force is positively related to original surface charge, which is consistent with Equation (2) (Figure 2F and Figure S3, Supporting Information). Droplet can be positively charged by contact electrification.^[^ ^44^, ^45^, ^46^ ^]^ Charging the droplet is beneficial for trapping, due to the contribution from electrostatic force (Note S2, Supporting Information). Meanwhile, the droplet charge also affects the asymmetry of the Janus charged droplet (Figure S4, Supporting Information).

### 2.3 Generality of Trapping Droplets

Note that the viscous force is negligible, owning to the extreme liquid repellency of the superamphiphobic surface. On the basis of force analysis, the roll‐off angle *α* for a droplet with mass (*m*) scales as follows (Note S4, Supporting Information):

(3) $$\alpha ∼\frac{\left(\varepsilon_{r}-1\right)\varepsilon_{0}\kappa^{4}Q^{2}}{m}$$

**Figure 3:**
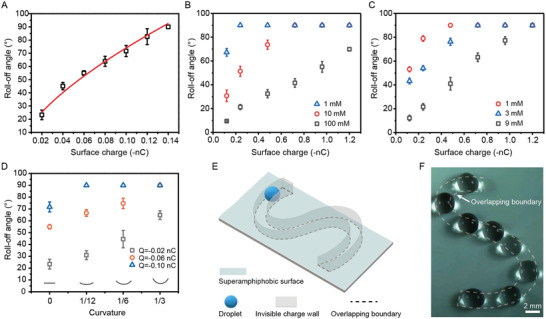
Generality of invisible charge wall at various titled angle, for different solution and in extensive boundary curvature. A) The roll‐off angle (*α*) of droplet as a function of surface charge. B) Generality of invisible charge wall for glucose solutions. C) Generality of invisible charge wall for NaCl solutions. D) The roll‐off angle of droplet as a function of boundary curvature. E) A sketch of the designed invisible charge wall with different curvature for guiding droplet transport. F) The time‐lapse trajectory of droplet transport along with the invisible charge wall.

Thus, the roll‐off angle of droplet at the invisible charge wall is determined by original surface charge, the curvature of overlapping boundary, dielectric property of droplet and droplet mass. The roll‐off angle is regulated by controlling the surface charge, which is assigned by the hydrodynamics of water impact (**Figure** **3**A and Equation (1)). We also demonstrate that the droplet trapping mediated by invisible charge wall is generic to different solutions and volumes (Figure 3B,C and Figure S5 and Table S1, Supporting Information). The invisible charge wall also manifests at diverse curvatures. The roll‐off angle increases with curvature, which is consistent with Equation (3). We can improve the roll‐off angle at low curvature by increasing the surface charge and droplet charge (Figure 3A,D and Figure S6, Supporting Information). Besides, we found that the length of the conductor does not affect the droplet trapping (Figure S7, Supporting Information). Note that the droplet can be trapped at the overlapping boundary with different curvatures. We designed the overlapping conductor with specific geometries to create complex pathways, to guide the droplet transport. For example, the invisible charge wall can be created into S shape pathway, which achieves well‐programmed droplet transport (Figure 3E,F and Movie S2, Supporting Information).

### 2.4 Applications of Invisible Charge Walls

**Figure 4:**
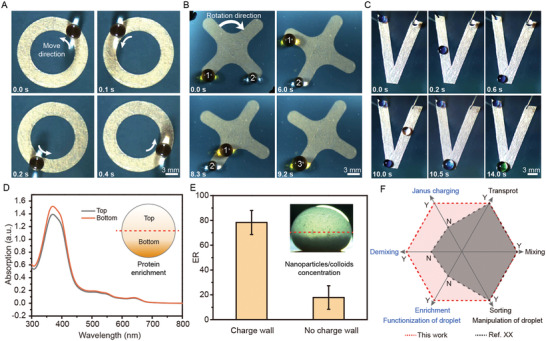
General applications of manipulating Janus charged droplet. A) A snapshot of circle invisible charge wall for droplet manipulation. The superamphiphobic surface is transparent so as to observe the trajectory of charge wall. B) A snapshot of cross invisible charge wall for droplet mixing. C) Bifurcated invisible charge wall for droplet fusing. D) Enrichment of protein in a single Janus charged droplet. E) The enrichment rate (ER) of nanoparticles in a single Janus charged droplet in 1 min. ER is defined as *R* = (*m*_1_ − *m*_0_)/*m*_0_ × 100%, where *m*_1_ is the mass of nanoparticles in the bottom part after enrichment, *m*_0_ is the mass of nanoparticles in the bottom part before enrichment. F) Qualitative comparison between invisible charge wall and previously reported droplet manipulation. “Y” and “N” represents yes and no, respectively.

To observe the location of invisible charge wall, we use transparent substrate to fabricate the droplet‐manipulated platform. Modeled conductor film is pasted underneath the charged superamphiphobic surface to construct charge wall. A circular invisible charge wall is prepared to limit the motion of droplet within a circle path (**Figure** **4**A and Movie S3, Supporting Information). A cross and V shaped invisible charge wall is designed to fuse and mix droplets (Figure 4B,C). The conventional trapping of droplet by wetting defect is irreversible and droplet cannot be further released.^[^ ^47^, ^48^ ^]^ In comparison, the invisible charge wall can be turned off by switching the conductor to the ground for completely discharging. Invisible charge wall achieves the reversibility between trap and release by keeping the conductor ungrounded and grounded (Figure 2A, Figure S8 and Movie S4, Supporting Information). The complete mobility control of droplet makes it feasible for extensive applications.

Besides tunable droplet mobility, the control of compositions in droplet is another essential prerequisite for developing next generation of microfluidics. The enrichment and separation in droplet are significant for synthesis, which could increase the speed of reactions and sensitivity of bioassays. We demonstrate that the unique Janus charged droplet manipulated by invisible charge wall is harnessed for biological and chemical applications. Here, we achieve in‐droplet electrophoresis that hemoglobin is concentrated in the manipulated Janus charged droplet (Figure 4D). Hemoglobin molecule is positively charged by adjusting pH value of the solution, so that the protein molecule can be attracted by the negative invisible charge wall (Figure S9, Supporting Information). Further, we show the rapid separation of nanoparticles in the Janus charged droplet (Figure 4E). A zeolitic imidazolate framework, ZIF‐8, is positively charged in the synthesis process (Figure S10, Supporting Information). The nanoparticles of ZIF‐8 in suspension occur fast precipitation at the invisible charge wall, compared with the normal state. These results are of significance because the invisible charge wall is feasible to accomplish in‐droplet demixing for any charged species. Taken together, our invisible charge wall demonstrates obvious advantages over conventional approaches in the aspect of functionalizing the manipulated droplets (Figure 4F).

## 3 Conclusion

In summary, we have designed an invisible charge wall to couple manipulation and functionalization of droplets. We also reveal that the invisible charge wall consists of the induced free charge on the overlapping conductor, which can be canceled so as to release the Janus charged droplet. The trapping force and asymmetry of the Janus droplet can be tuned by controlling the original surface charge on superamphiphobic surface. The generality of various liquids and different curvature of overlapping boundary enables us to design trajectory for liquids manipulation. It is worth noting that the biggest advantage of invisible charge wall lies in the functionalization of the manipulated droplet, allowing a rapid enrichment and separation of the charged composition within droplets. This development potentially contributes to the speed of synthesis and sensitivity of bioassays. From a broader perspective, the invisible charge wall provides a versatile microfluidic platform for applications from in‐droplet reaction and separation to detection.

## 4 Experimental Section

### Materials

The following chemicals were obtained from Chron Chemicals: NaCl (AR, 99.5%), citric acid (AR), D‐glucose monohydrate (AR), zinc acetate dihydrate (AR), sodium hydroxide (AR, 98%). Tartrazine (85%), tetraethyl orthosilicate (98%), and ammonia solution (28% in water) were purchased from Aladdin Industrial Corporation. Indigo Carmine (≥70%) and 2‐methylimidazole (98%) were obtained from Adamas beta. Trichloro (1H,1H,2H,2H‐Perfluorooctyl) Silane (97%) was purchased from Sigma Aldrich. Hemoglobin was obtained from Bioss. Deionized water with a resistivity of 18.5 MΩ cm was used for the measurement.

### Preparation of Droplet Manipulation Platform

Glass slides (Deckglaser glass coverslips) were first coated with candle soot, and then placed in a desiccator together with tetraethoxysilane (1 mL) and ammonia solution (1 mL). The desiccator was closed, and the vacuum was maintained for 24 h. Then, the carbon soot core was removed through annealing at 600 °C for 2 h in air. The annealed samples were treated with air plasma for 10 min using a plasma cleaner (Tonson Tech Automation Equipment) at high power followed by another chemical vapor deposition sequence of trichloro (1H,1H,2H,2H‐Perfluorooctyl) Silane (100 µL) in vacuum for 6 h to lower the surface energy. The conductive copper tape with different shapes was prepared by laser cutting and used for overlapping with superamphiphobic surface. The thickness of conductive copper tape was 0.065 mm and the width was 3 mm.

### Droplet Motion Recording

Droplets of deionized water, tartrazine solution, sodium hydroxide solution, and indigo carmine solution were used. The droplets were deposited from metal needle tips connecting with a micro‐injection pump (Longer, LSP01‐1A). Surface with circular path was placed on a circular shaker (NuoMi, NMYC‐200) at a speed of 130 rpm. Surface with straight, S, cross and V shaped path were tilted with an angle of 10°. The droplet motions were recorded by a high‐speed camera (Photron Mini UX50) at a speed of 2000 fps. Videos were analyzed by using Tracker and PFV software. The relative humidity was kept constant at ≈40 RH%, and the room temperature was 20 °C.

### The Measurement of Electrical Property

The superamphiphobic surface by impacting with water drop at different heights was charged. The charge of impacted droplet was measured by a Faraday cup connected to an electrometer (ESD‐China, EST111). The absolute value of droplet charge was equivalent to the surface charge for each impact. Since the total surface charge on superamphiphobic surface was the sum of charges generated at different locations, several times at different positions to adjust the surface charges were impacted. At the same time, the surface potential was measured by an electrostatic voltmeter with a non‐contacting probe (Trek Inc., model 341B). Before each charging, the superamphiphobic surface was neutralized by using an ionizing air blower (DR. SCHAEIDER PC, Model SL‐001). The relative humidity was kept constant at ≈40 RH%, and the room temperature was 20 °C.

To measure Janus charge precisely, a special metal needle connected with plastic pipette to absorb liquid was used. The needle was 80 mm in length and 0.84 mm in inner diameter to ensure its volume large enough to avoid the touch between liquid and plastic parts. The needle and pipette were discharged by ion wind in advance before using. A neutral droplet (40 µL) was placed onto charged superamphiphobic surface. The polarization was completed in seconds. Half part of the droplet from the upper layer was pipetted slowly and it was spit out to Faraday cup rapidly to measure its charge (Figure S11, Supporting Information). The measurement of the left was conducted in the same way.

### Protein Enrichment and Nanoparticles Separation

5 mg mL^−1^ of hemoglobin solution was used for protein enrichment. The protein solution was adjusted to pH = 4 in order to make the hemoglobin positively charged. Hemoglobin can keep its biological activity at that pH value. The Zeta potential of the protein solution was measured by using Zeta Sizer (Malvern, ZEN3690). Hemoglobin solution (40 µL) was released on the charged superamphiphobic surface and trapped at the overlapping boundary. The upper and bottom layer were separated with equal volume after 1 min. Repeating the above steps for 5 times. The collected solution of upper and bottom parts were diluted to 2 mL for concentration measurement, respectively. The concentration was measured by UV–vis (Shimadu, UV‐2550).

14.3 mg mL^−1^ of zeolitic imidazolate framework (ZIF‐8) nanoparticles was used for nanoparticles separation. ZIF‐8 was synthesized by method reported before.^[^ ^49^ ^]^ Zinc acetate dihydrate (0.12 g) was dissolved in deionized water (10 g). A solution consisting of 2‐methylimidazole (0.11 g) and deionized water (10 g) was added into the Zn‐based solution and stirred 12 h. The resultant particles were separated from the solution by centrifugation at 10 000 rpm and washed with deionized water. This procedure was repeated for three times. ZIF‐8 powder was dried in a vacuum oven. The obtained ZIF‐8 nanoparticles were redispersed in 4 mL of deionized water. Then 60 µL of the suspension was released onto the charged and uncharged superamphiphobic surface, respectively. The separation operation was repeated for 10 times and obtained 300 µL of upper and bottom of droplet, respectively. The mass of ZIF‐8 was measured after drying. The enrichment rate (ER) was calculated using the following equation: ER = (*m*_1_ − *m*_0_)/*m*_0_ × 100%, where *m*_1_ is the mass of ZIF‐8 in the bottom part after enrichment, *m*_0_ is the mass of ZIF‐8 in the bottom part before enrichment.

## Conflict of Interest

The authors declare no conflict of interest.

## Author Contributions

Q.S. designed research; Q.S., X.H., and B.X. performed research; Q.S., X.H., and S.L. analyzed data; and Q.S., X.H., X.D., and S.Z. wrote the paper.

## Supporting information

Supporting Information

Supplemental Video 1

Supplemental Video 2

Supplemental Video 3

Supplemental Video 4

## Data Availability

The data that support the findings of this study are available from the corresponding author upon reasonable request.
