# Supplementary material for: Janus Charged Droplet Manipulation Mediated by Invisible Charge Walls
Source: Adv Sci (Weinh). 2022 Oct 6;9(33):2204382. doi: 10.1002/advs.202204382 (PMC9685436; doi:10.1002/advs.202204382)
Supplement: Supplementary file 1 — Supporting Information [file ADVS-9-2204382-s004.pdf]

Supplementary Information for
Janus Charged Droplet Manipulation Mediated by Invisible Charge Walls

Qiangqiang Sun[a]*, Xuanming Hu[a], Boran Xu[a], Shiji Lin[b], Xu Deng[c]*, Shaobing Zhou[a]*

*Qiangqiang Sun, *Xu Deng, *Shaobing Zhou.
Email: qiangqsun@swjtu.edu.cn; dengxu@uestc.edu.cn; shaobingzhou@swjtu.edu.cn

This PDF file includes:

Supplementary text
Figures S1 to S9
Tables S1
Legends for Movies S1 to S4
SI References

Other supplementary materials for this manuscript include the following:

Movies S1 to S4

Supplementary Information Text

Note S1. The simulation of surface potential.

The electric potential distribution of superamphiphobic surface with a charge area is modeled by COMSOL Multiphysics V5.3a. The simulation space and boundaries are shown below. The surface has a width of 24 mm and length of 60 mm. In model one and model two, the charged area with 0.11 nC are a circle of 2.4 mm-radius and a rectangle of 5 mm×30 mm, respectively. The electric potential V is governed by the Poisson's equation: $\vec{\nabla}\cdot\vec{\nabla}V = -\frac{\rho_S}{\varepsilon_0\varepsilon_{rs}}$, where ρ_S is the surface charge density which is calculated by the total amount of charges and the charged area. ε_0=1 and ε_{rs}=4.5 is the dielectric constant of vacuum and of surface. The electric potential is defined from the electric field intensity *E* by the following equation: $\vec{\mathrm{E}} = -\vec{\nabla}V$.

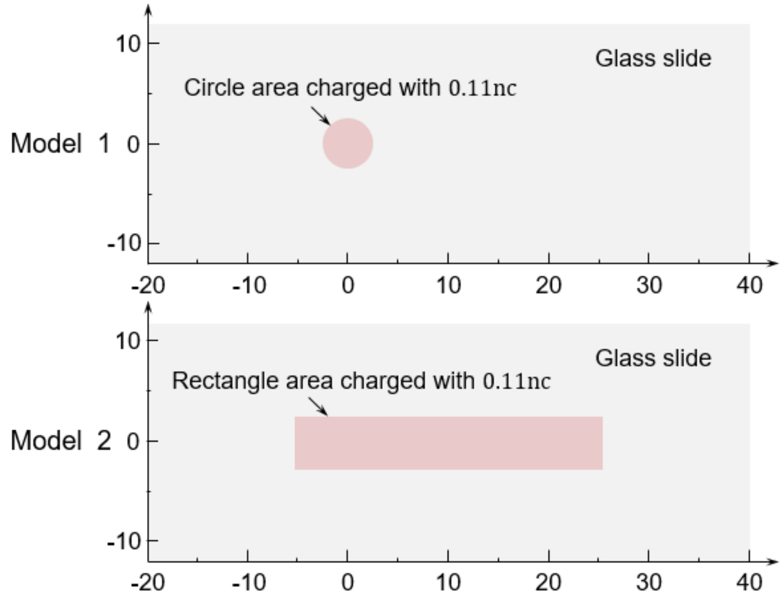

Geometry of electrostatic model.

Note S2. Analysis of trapping force on droplets.

The invisible charge wall can polarize the droplet and generate an electric force to stop the droplet motion. When a droplet is trapped at the overlapping boundary, the accumulated charge amount near the trapped droplet is *σA*. Here, *A* is the area near the overlapping boundary. The invisible charge wall gives a trapping force $F = \frac{k_e\sigma A Q_p}{r^2}$, where *r* is the nearest distance from the superamphiphobic surface to droplet, k_e is the Coulomb constant, Q_p is the polarization charge on droplet induced by the *σA*, and it can be written as $Q_p = \nabla(\varepsilon_r - 1)\varepsilon_0 E(1)$. Here, ∇ is the delta vector operator, ε_r is the relative dielectric constant of the droplet, ε_0 is the dielectric constant of empty space and *E* is the electric field produced by *σA*. The electric field can be expressed as $E = \frac{k_e\sigma A}{r^2}$. Therefore, the trapping force becomes:

$$F = \nabla\frac{k_e^2}{r^4}(\varepsilon_r - 1)\varepsilon_0\sigma^2 A^2 \qquad (1)$$

We have known that the charge density at the overlapping boundary is proportional to the boundary curvature and original surface charge: $\sigma\sim\kappa^2 Q$. So, the trapping force *F* can scales as:

$$F\sim(\varepsilon_r - 1)\varepsilon_0\kappa^4 Q^2 \qquad (2)$$

If the droplet carries an initial charge Q_0, the trapping force will increase due to the additional electrostatic force. After adding an electrostatic force, equation (1) becomes:

$$F = \nabla \frac{k_e^2}{r^4}(\varepsilon_r - 1)\varepsilon_0\sigma^2 A^2 + \frac{k_e \sigma A Q_0}{r^2} \quad (3)$$

Note S3. Trapping force measurement.

We use a homemade set-up to measure the trapping force at the invisible charge wall. The device is shown in Figure S1. The trapping force (*F*) has the relationship of $3EI = \frac{FL^3}{\Delta L}$, where *E* is the elastic modulus of cantilever beam, *I* is the inertia moment of a cantilever beam section and *L* is termed as the length of cantilever beam. We define *3EI* as *K*, which is a constant related to width and thickness of cantilever beam. The magnitude of *K* was calibrated by placing droplets with different volumes at the end of the horizontal cantilever beam. The cantilever beam is stainless steel film with a width of 10 mm and a thickness of 0.02 mm. The deformation (*ΔL*) of the cantilever beam caused by droplet gravity is recorded by a laser displacement meter. According to the result, *K* is equal to $12*10^3$ μN·mm^3.

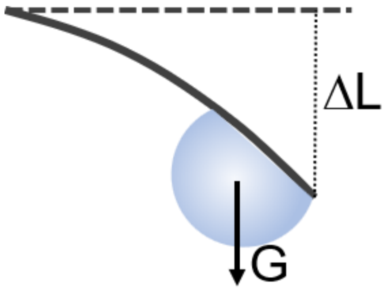

Schematic diagram of K calibration process.

Then, a 10-cm-long cantilever beam was placed vertically to measure droplets' trapping force on substrate. We connected a PTFE rod of length 5 mm at the end of the stainless steel cantilever beam to contact with the droplet in order to avoid the adhesion between stainless steel and droplet. Thus, the variation of *ΔL* is recorded by the laser displacement meter in real time and *F* is calculated by using equation of $F = \frac{\Delta L \cdot K}{L^3}$.

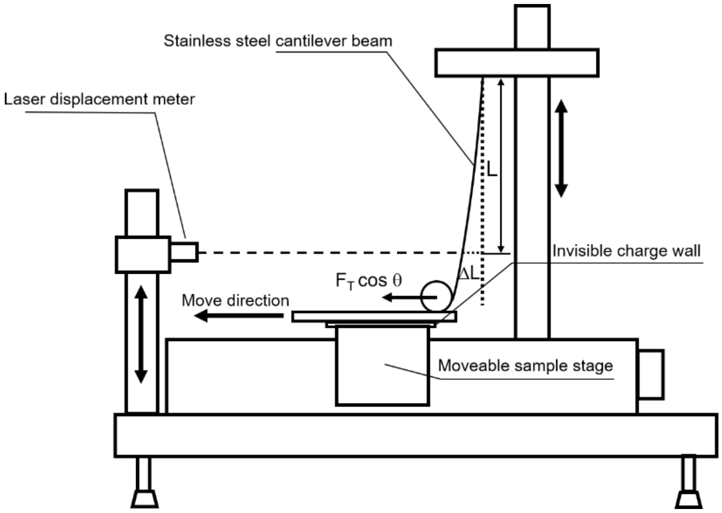

Sketch of the trapping force measurement. A droplet is placed at the invisible charge wall. The substrate is mounted on a linear stage driven by a step motor. A laser displacement meter is used to record the deflection of the cantilever.

Note S4. Roll-off angle of droplets.

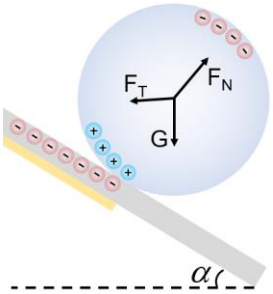

Free body diagram of droplet at overlapping boundary

We analyze the forces on the trapped droplet at overlapping boundary with a titled angle of *α*. The viscous force is negligible, because of the extreme liquid repellency of the superamphiphobic surface. The droplet is mainly influenced by the gravitational and trapping force. Thus, we give equation below based on simple force analysis:

$$mg\sin\alpha = \nabla \frac{k_e^2}{r^4}(\varepsilon_r - 1)\varepsilon_0\sigma^2 A^2 \qquad (4)$$

m is the mass of droplet, *g* is the gravitational acceleration and *α* is the roll-off angle. Equation (4) also can be written as:

$$\alpha = \sin^{-1}\nabla \frac{k_e^2}{mgr^4}(\varepsilon_r - 1)\varepsilon_0\sigma^2 A^2 \qquad (5)$$

So, the roll-off angle *α* scales as:

$$\alpha \sim \frac{(\varepsilon_r - 1)\varepsilon_0\kappa^4 Q^2}{m} \qquad (6)$$

As shown in Figure S5, as the droplet mass increases, the angle becomes smaller.

If the droplet carries an initial charge Q_0, the roll-off angle will also increase due to the additional electrostatic force. After adding an electrostatic force, equation (5) becomes:

$$\alpha = \sin^{-1}\left[\nabla \frac{k_e^2}{mgr^4}(\varepsilon_r - 1)\varepsilon_0\sigma^2 A^2 + \frac{k_e\sigma A Q_0}{mgr^2}\right] \qquad (3)$$

Obviously, for the same surface charge, droplets with initial charge will have a greater roll-off angle. (Figure S8)

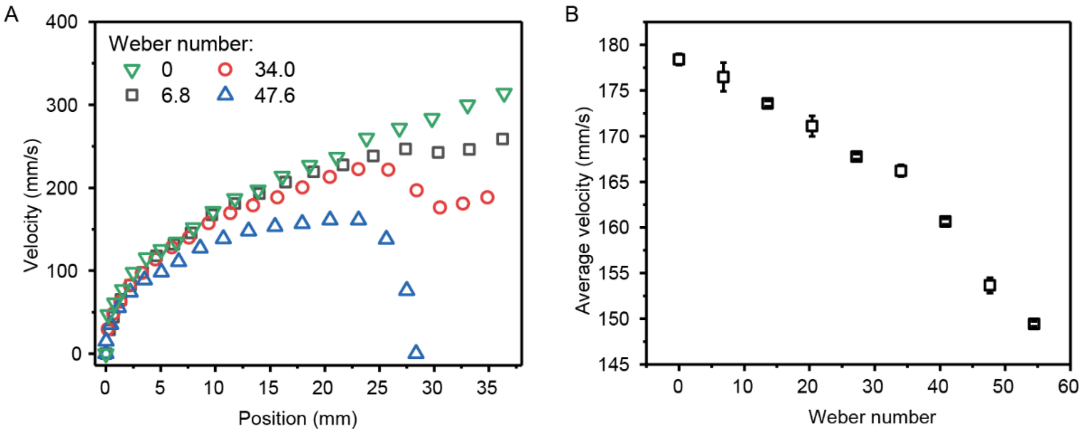

Figure. S1. Velocity of droplet in the overlapping area. Instantaneous (A) and average (B) velocities of droplets moving in a straight line in the overlapping area at various Weber number. Error bars denote the standard error value from three measurements.

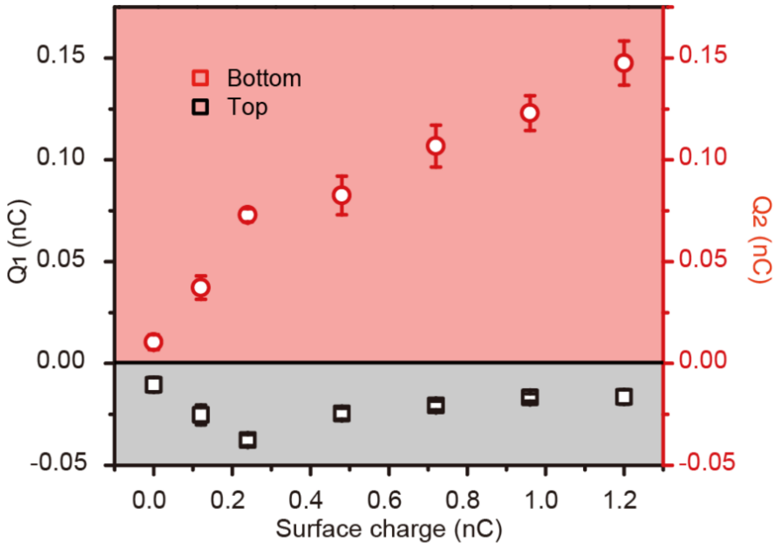

Figure. S2. The charge value of Janus charged droplet as a function of surface charge. The droplet is separated to bottom and top parts with equal volume for the charge measurement. Error bars denote the standard error value from three measurements.

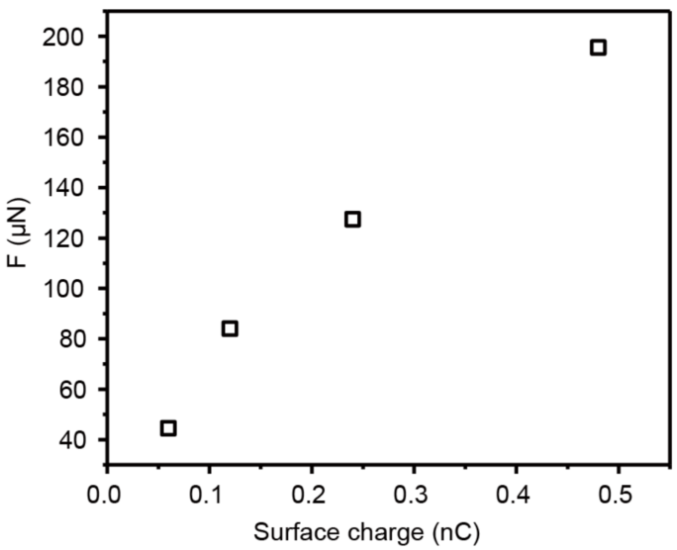

Figure. S3. The trapping force as a function of surface charge corresponding to Figure .2F.

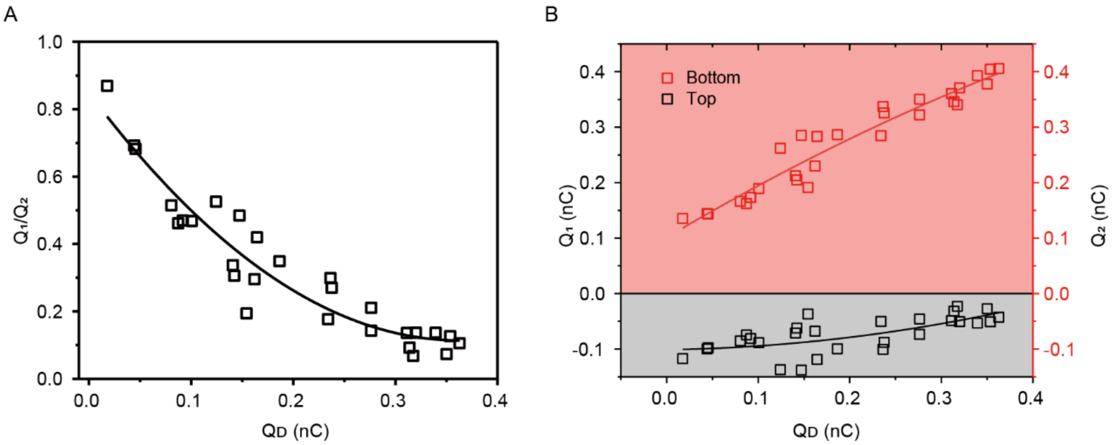

Figure. S4. Affection of droplet charge on Janus droplet asymmetry. A, the ratio of negative and positive charges in a droplet as a function of droplet charge. B, negative and positive charges in droplets as a function of droplet charge.

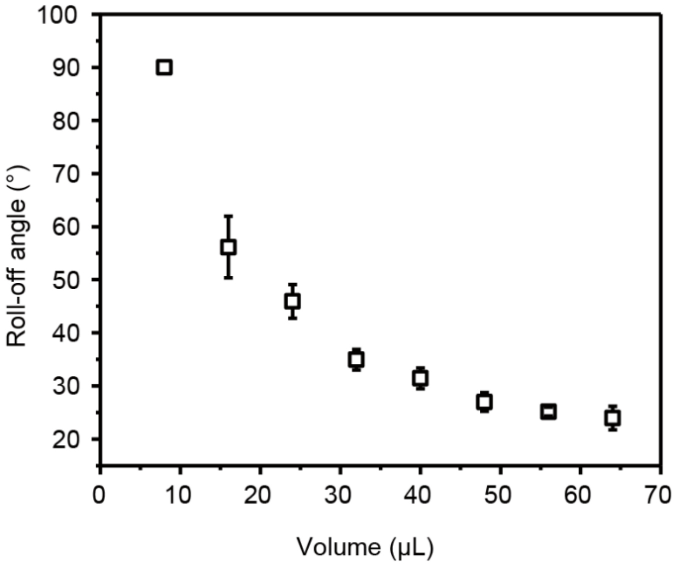

Figure. S5. Evolution of the roll-off angle as a function of droplets' volume. Error bars denote the standard error value from three measurements.

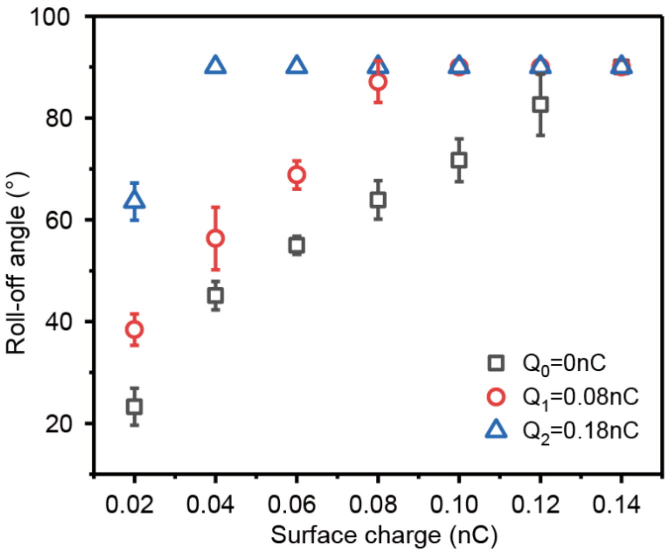

Figure. S6. Roll-off angles of droplets with different initial charges as a function of surface charge. Error bars denote the standard error value from three measurements.

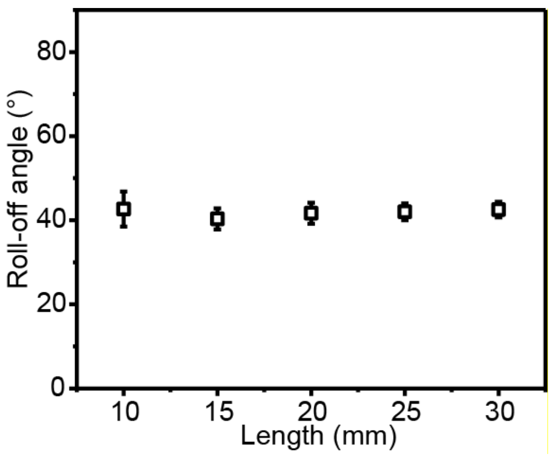

Figure. S7. The effect of the conductor length on droplet trapping when the surface charge is equal to 0.06 nC.

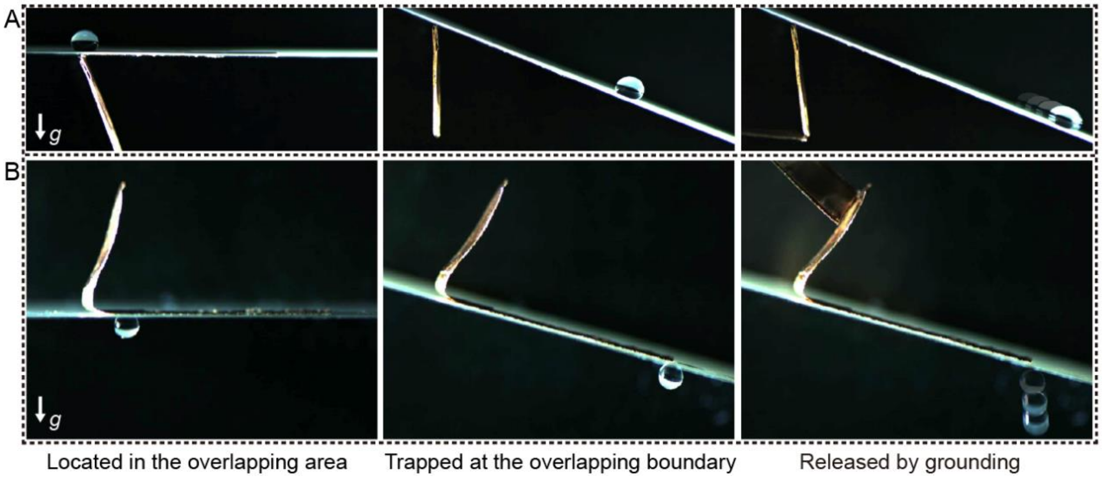

Figure. S8. Trapping switch. Droplets are trapped at the invisible charge wall and released at the overlapping boundary when the conductive film get grounded.

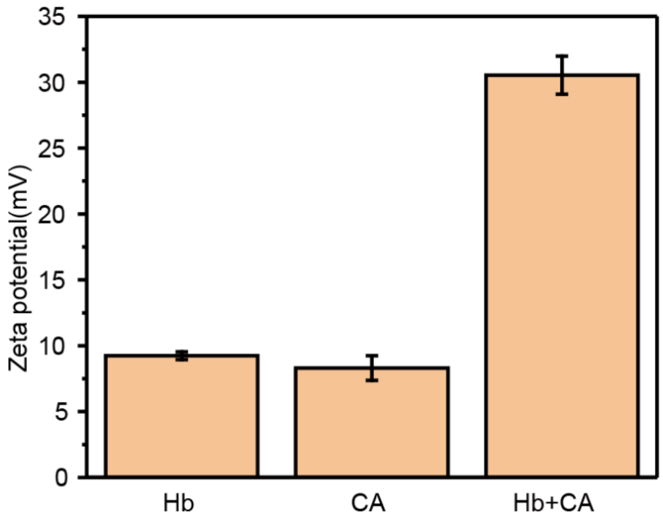

Figure. S9. Zeta potential of hemoglobin (Hb) solution, citric acid (CA) solution and solution of mixed citric acid and hemoglobin.

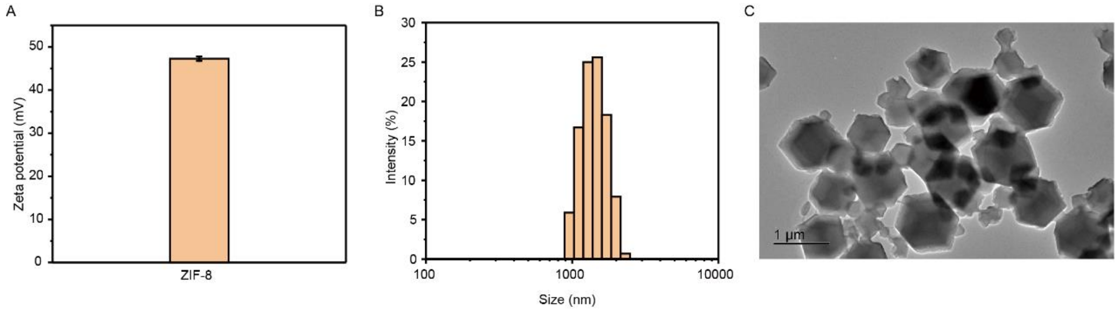

Figure. S10. Characterizations of ZIF-8 nanoparticles. Zeta potential (A), size distribution (B) and TEM image (C) of ZIF-8 nanoparticles.

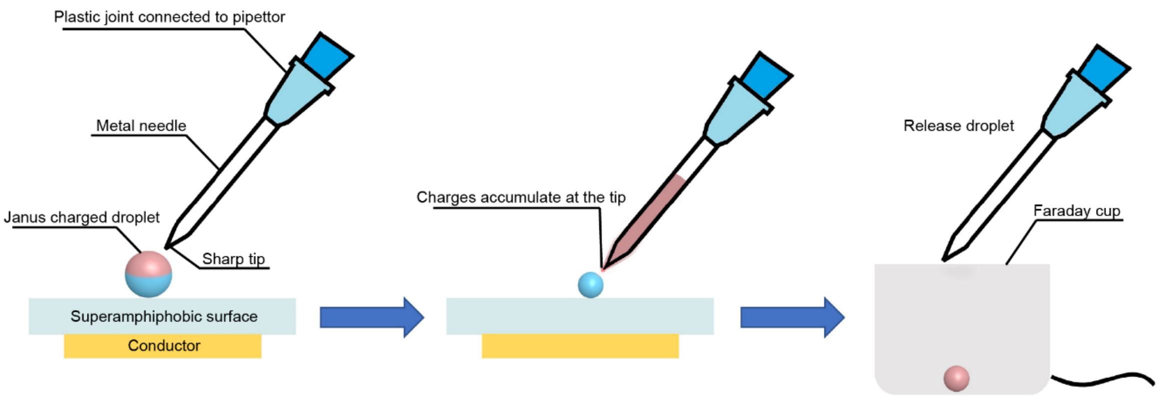

Figure. S11. Schematic of the measurement for charges in Janus charged droplet.

Table S1. Physical properties of DI water, NaCl and glucose solution at room temperature.

Liquids	Relative dielectric constant [ε_r]	Surface tension [mN/m]	Density [g/cm^3]	Superamphiphobic surface SCA [°]	Roll-off angle [°]
DI water	81	72.1	1.00	166±1	1±1
0.1 M NaCl	78.7	72.2	1.005	162±1	1±1
0.3	74.6	72.5	1.012	162±1	1±1
0.5	73.4	73.1	1.0195	162±1	1±1
0.7	72.0	73.4	1.023	162±1	1±1
10 wt% Glucose solution	71.74	73.1	1.11	164±1	1±1
20 wt% Glucose solution	68.71	73.2	1.25	164±1	1±1
30 wt% Glucose solution	64.11	74.8	1.43	164±1	1±1
40 wt% Glucose solution	57.83	77.2	1.67	163±1	1±1

Movie S1 (separate file). Different motion state of droplet on charged superamphiphobic surface contacting with (bottom) and without (up) conductor at an inclination angle of 15 °.

Movie S2 (separate file). The designed invisible charge wall for guiding droplet manipulation.
Movie S3 (separate file). The separation of nanoparticles in a single Janus charged droplet.
Movie S4 (separate file). The droplet release from the invisible charge wall realized by switching the conductor to the ground.

SI References

1. R. Feynman, R. Leighton, M, Sands, Mainly Electromagnetism and Matter, (Addison-Wesley, 1979), Vol. 2 of The Feynman Lectures on Physics.
